# Knowledge, attitude and associated factors among primary school teachers regarding refractive error in school children in Gondar city, Northwest Ethiopia

**DOI:** 10.1371/journal.pone.0191199

**Published:** 2018-02-15

**Authors:** Abiy Maru Alemayehu, Gizchewu Tilahun Belete, Nebiyat Feleke Adimassu

**Affiliations:** Department of Optometry, College of Medicine and Health Sciences, University of Gondar, Gondar, Ethiopia; The Chinese University of Hong Kong, HONG KONG

## Abstract

**Introduction:**

Refractive error is an important cause of correctable visual impairment in the worldwide with a global distribution of 1.75% to 20.7% among schoolchildren. Teacher’s knowledge about refractive error play an important role in encouraging students to seek treatment that helps in reducing the burden of visual impairment.

**Objective:**

To determine knowledge, attitude and associated factors among primary school teachers regarding refractive error in school children in Gondar city.

**Methods:**

Institution based cross-sectional study was conducted on 565 primary school teachers in Gondar city using pretested and structured self-administered questionnaire. For processing and analysis, SPSS version 20 was used and variables which had a P value of <0.05 in the multivariable analysis were considered as statistically significant.

**Result:**

A total of 565 study subjects were participated in this study with a mean age of 42.05 ± 12.01 years. Of these study participants 55.9% (95% CI: 51.9, 59.8) had good knowledge and 57.2% (95% CI: 52.9, 61.4) had favorable attitude towards refractive error. History of spectacle use [AOR = 2.13 (95% CI: 1.32, 3.43)], history of eye examination [AOR = 1.67 (95% CI: 1.19, 2.34)], training on eye health [AOR = 1.94 (95% CI; 1.09, 3.43)] and 11–20 years of experience [AOR = 2.53 (95% CI: 1.18, 5.43)] were positively associated with knowledge. Whereas being male [AOR = 2.03 (95% CI: 1.37, 3.01)], older age [AOR = 3.05 (95% CI: 1.07, 8.72)], 31–40 years of experience [AOR = 0.23 (95% CI: 0.07, 0.72)], private school type [AOR = 1.76 (95% CI: 1.06, 2.93)] and 5^th^ -8^th^ teaching category [AOR = 1.54 (95% CI: 1.05, 2.24)] were associated with attitude.

**Conclusion:**

Knowledge and attitude of study subjects were low which needs training of teachers about the refractive error.

## Background

A refractive error (RE) is an error in the focusing of light on the retina. it is one of the most common ocular conditions, and uncorrected refractive error(URE) is a major public eye health challenge [1]. Worldwide, URE is the leading cause of visual impairment and second leading cause of blindness[2]. The pattern of RE among children has considerable variation from one geographic location to the other which is attributable to hereditary and environmental factors [3]. According to world health organization, refractive error is responsible for 42% and 3% of visual impairment and blindness respectively in the world [2]. The magnitude of refractive error across the world among schoolchildren ranges from 1.75% to 20.7% [4–10].

Undetected and untreated vision problems interfere with the ability to perform to one's full learning potential and place a greater socioeconomic burden on the society. To lessen this impact, via early detection and referring school children to eye care services by school teachers, exploration of knowledge and attitude in a population is very important [11,12].

Knowledge of primary school teachers towards RE plays an important role in encouraging children to seek treatment for their eye problems as well as to enhance eye health seeking behavior [13]. In addition to this, it helps to minimize the burden of visual impairment due to URE [14,15]. Having this importance the level of knowledge in different studies ranges from 1% to 89% [15–20]. The attitude of primary school teachers towards RE also has a positive impact on prevention of visual impairment from RE in school children [21]. In contrast, negative attitude towards RE management options can result in psychological impact as revealed in different studies [22,23].

Children spent most of their time in the school and it is possible for teachers to access them easily. This creates a good opportunity for the teachers to identify RE in the early stage. Early detection and management of the RE is very much important to prevent visual impairment, blindness, and its sequela. Therefore the knowledge and attitude towards RE among school teachers play a paramount role to detect any manifestations of RE in school children. However, having this role, baseline data about school teachers’ knowledge and attitude towards RE is limited in the study area. Therefore, this study determines the knowledge, attitude and associated factors among primary school teachers towards a refractive error in school children so that it will help respective stakeholders to act accordingly.

## Methods and materials

### Study design and population

An Institution-based cross-sectional study was conducted on primary school teachers in Gondar city, between April 20 and May 23, 2017. A total of 1644 primary school teachers from 64 primary schools participated in the study.

### Sample size determination

Due to the lack of published literature investigating the knowledge and attitude of primary school teachers regarding a refractive error in Ethiopia as well as in the study area, the present study calculated the maximum possible sample size. To achieve this, 50% proportion was considered, for which, the sample size came to 586. The margin of error 3.5%, the 95% confidence level and 10% for the non—response rate was considered during the sample size calculation. These 586 teachers were from the 17 randomly selected primary schools.

### Sampling technique and procedures

To ensure representativeness, samples were taken from about 25% of the total 64 primary schools. Seventeen schools out of 64 schools were selected using simple random sampling method after a list of schools obtained from the Gondar city administrative educational office. In the selected 17 schools (10 government and 7 private schools), there were a total of 587 teachers. So, all of these teachers were included in the study.

The study was conducted after ethical clearance obtained from the ethical review board of the University of Gondar. Officials at the school of medicine, Gondar city administrative education office and selected schools were communicated through formal letters. Each participating school was visited a week before the data collection day, and permission to conduct the study was taken from the schools. Written informed consent was obtained from each study participant. Participants were informed about the objective of the study and they were given full right to discontinue or refuse to participate in the study.

### Operational definition

#### Knowledge

The knowledge of refractive error was assessed using a 18 point scale. There were eleven multiple choice questions that carried a total of 18 correct responses. Each correct response was given a score of 1 and a wrong response a score of 0. Total points to be scored were 18 and the minimum score was 0. A related study was used for classification of study participants’ knowledge level. With a score of mean and above meant a good knowledge and poor knowledge for a score less than mean[24].

**Good knowledge**: Individuals who responded the mean (7.92) and above of the total knowledge questions had good knowledge about refractive errors in school children.

**Poor knowledge**: Individuals who responded below the mean (7.92) of the total knowledge questions had poor knowledge about refractive errors in school children.

#### Attitude

The attitude was measured by eight questions put on Likert’s scale. The questions on Likert’s scale had positive and negative responses that ranged from strongly agree, agree, neutral, disagree and strongly disagree. The scoring system used with respect to participant’s responses was as follows: strongly agree 5, agree 4, neutral 3, disagree 2, and strongly disagree 1. The responses were summed up and a total score was obtained for each respondent. The mean was calculated and those who scored above the mean value had favorable attitude and the ones who scored less than the mean value had an unfavorable attitude towards a refractive error in school children [24].

**Favorable attitude**: Respondents who answered greater than or equal to the mean (30.22) attitude questions had a favorable attitude.

**Unfavorable attitude**: Respondents who answered below the mean (30.22) attitude questions had an unfavorable attitude.

### Data collection tools, procedures, and personnel

Pre-tested and structured self-administered questionnaire was employed to collect data. The questionnaire was developed from literature [16]. It was translated into Amharic version (local language) from English version and then back to the English version for consistency and ease of the interview. Trained eight optometrists participated in the data collection.

### Data quality control

The pretest was done out of the study area to validate the questionnaire by taking 5% of the sample size. After doing the pre-test, necessary modifications were made accordingly before the actual data collection was performed. Training was given to data collectors on how to collect the data. On the field work, the supervisor has closely followed the day-to-day data collection process and ensure internal consistency of the collected data. Finally, 5% of the collected samples were checked for completeness by the principal investigator in each day.

### Data processing and statistical analysis

After cleaning and coding, the data were entered into EpiData version 3.1 and were exported and analyzed using SPSS version 20. Analysis was done by the principal investigator using the same computer package. The variables that were found with P<0.2 at binary logistic regression were entered to multivariable analysis in which multiple logistic regression was used and those variables with a p-value less than 0.05 were considered as statically significant.

## Results

### Socio-demographic characteristics of the study participants

A total of 565 primary school teachers who were working in Gondar city took part in the study with a response rate of 96.3%.

Among that 52.9% (299) were females. The mean age of the study participants was 42.05 years (SD = ± 12.01 years). Most of the study participants (78.6%) were orthodox Christian. Primary school teachers who earned diploma accounts 75.7%. Of the total participants, 63.4% were married. On the other hand, 416 (73.6%) teachers were enrolled in governmental schools in which 240 (57.7%) of them taught from grade 5^th^ to 8^th^ (see Table 1).

**Table 1:** Socio-demographic characteristics of study participants in Gondar city, Northwest Ethiopia, 2017.

Variables	Frequency	Percent
Age category		
21–30 years	155	27.4
31–40 years	106	18.8
41–50 years	107	18.9
51–70 years	197	34.9
Sex		
Female	299	52.9
Male	266	47.1
School type		
Government	416	73.6
Private	149	26.4
Religion		
Orthodox	444	78.6
Muslim	74	13.1
Protestant	47	8.3
Educational status		
Certificate	40	7.1
Diploma	428	75.7
Degree	97	17.2
Monthly income in birr		
1000–2180	39	6.9
2181–3361	147	26.0
3362–4541	194	34.3
4542–5722	147	26.0
>5723	38	6.8

### Attitude and level of knowledge towards refractive error among study participants

Out of 565 study participants 316 (55.9%) (95% CI: 51.9, 59.8) had good knowledge. Among study participants, who had poor knowledge, 82(14.5%) didn’t know what refractive error is. Blurring of vision was the most known symptoms by study participants.

Of the total respondents, 323 (57.2%) (95% CI: 52.9, 61.4) had a favorable attitude towards refractive error. Among study participants, 285 (50.4%) agreed that non-eye care professionals can perform vision screening in children. Most study participants (89.6%) agreed that complication of refractive error can be prevented (see Table 2).

**Table 2:** Proportion of the level of knowledge and attitude towards refractive error among study participants in Gondar city, Northwest Ethiopia, 2017.

Variables	Frequency	Percent
Level of knowledge (n = 565)		
**Good**	316	55.9
**Poor**	249	44.1
Attitude (n = 565)		
**Favorable**	323	57.2
**Unfavorable**	242	42.8

Respondents who used information (84.60%) like mass media, health personnel and colleagues were found to have good knowledge (60.88%) and favorable attitude (54.39%) towards refractive error.

### Factors associated with the level of knowledge towards refractive error

In the bi-variable analysis being older age, Muslim, years of experience, higher level of educational status, being married, high income, working in private school, previous eye examination, use of spectacle, previous training on eye health and family spectacle use show association with level of knowledge towards refractive error among primary school teachers. Whereas in multivariable analysis years of experience, previous history of eye examination, history of spectacle use and previous training on eye health were found to be significantly associated with knowledge (see Table 3).

**Table 3:** Factors associated with knowledge of study participant regarding a refractive error in school children in Gondar city, Northwest Ethiopia, 2017.

Variables	Knowledge			
	Good	Poor	COR (95% CI	AOR (95% CI)
Family spec use*				
No	230	195	1.00	
Yes	86	54	1.35(0.91, 1.99)	
School type*				
**Government**	239	177	1.00	
**Private**	77	72	0.79(0.54, 1.15)	
Religion*				
**Orthodox**	256	188	1.00	
**Muslim**	39	35	0.82(0.49,1.34)	
**Protestant**	21	26	0.59(0.32,1.09)	
Spectacle usage				
**No**	174	188	1.00	1.00
**Yes**	142	61	2.52(1.75, 3.62)	2.13(1.32,3.43)**
Monthly income in birr*				
**1000–2180**	18	21	1.00	
**2181–3361**	74	73	1.83(0.58, 2.40)	
**3362–4541**	110	84	1.53(0.77, 3.05)	
**4542–5722**	90	57	1.84(0.90, 3.75)	
**>5723**	24	14	2.00(0.80, 4.98)	
Previous eye exam				
**No**	141	143	1.00	
**Yes**	175	106	2.01(1.12,3.59)	1.67(1.19, 2.34)**
Marital status*				
**Single**	57	62	1.00	
**Married**	218	140	1.69(1.12, 2.57)	
**Divorced**	28	28	1.09(0.78, 2.05)	
**Widowed**	13	19	0.74(0.34, 1.64)	
Educational status*				
**Certificate**	26	14	1.00	
**Diploma**	229	199	0.62(0.32, 1.22)	
**Degree**	61	36	0.91(0.42,1.97)	
Training on eye health				
No	263	228	1.00	
Yes	53	21	2.19(1.28, 3.74)	1.94(1.09, 3.43)**
Age in years*				
21–30 years	75	80	1.00	
**31–40 years**	49	57	0.92(0.56, 1.50)	
**41–50 years**	58	49	1.26(0.77, 2.07)	
**51–70 years**	134	63	2.27(1.47, 3.50)	
Years of experience				
**1–10 years**	59	79	1.00	1.00
**11–20 years**	81	70	1.55(0.97, 2.47)	2.53(1.18, 5.43)**
**21–30 years**	81	54	2.01(1.24, 3.25)	2.48(0.87, 7.02)
**31–40 years**	95	46	2.77(1.69, 4.50)	2.16(0.67, 2.00)

* Non-significant,

** P-value <0.05

COR = Crude odds ratio, AOR = Adjusted odds ratio

Table 3 showed that the odds of good knowledge regarding refractive error among study participants who had the previous history of spectacle were two times greater than the odds of good knowledge for those subjects who didn’t use spectacle [AOR = 2.13 (95% CI: 1.32, 3.43)]. In addition to this, the odds of good knowledge regarding refractive error among study participants with the previous history of eye examination were 1.67 times larger than the odds of good knowledge for those subjects with no history of eye examination [AOR = 1.67 (95% CI: 1.19, 2.34)]. The odds of good knowledge regarding refractive error among study participants who had previous training on eye health were two times greater than the odds of good knowledge for study subjects with no history of training on eye health [AOR = 1.94 (95% CI; 1.09, 3.43)]. The odds of good knowledge regarding refractive error among subjects with 11–20 years of experience were 2.53 times higher than the odds of good knowledge among subjects who had 1–10 years of experience [AOR = 2.53 (95% CI: 1.18, 5.43)].

### Factors associated with attitude of study participants towards refractive error

In the bi-variable analysis age, religion, many years of experience, being divorced, working in private school, 5^th^ -8^th^ teaching category, sex being female and family spectacle use show association with a favorable attitude towards refractive error among primary school teachers. Whereas in multivariable analysis many years of experience, working in private school, being older age, being male and 5^th^ -8^th^ teaching category were found to be significantly associated with attitude (see Table 4).

**Table 4:** Factors associated with attitude among primary school teachers regarding refractive error in school children in Gondar town, Northwest Ethiopia, 2017.

Variables	Attitude			
	Favorable	Unfavorable	COR (95% CI)	AOR (95% CI)
Sex				
**Female**	136	163	1.00	
**Male**	187	89	2.84(2.00, 4.02)	2.03(1.37, 3.01)***
School type				
**Government**	215	201	1.00	
**Private**	108	41	2.46(1.64, 3.70)	1.76(1.06, 2.93)**
Religion*				
**Orthodox**	242	202	1.00	
**Muslim**	56	18	2.59(1.48, 4.56)	
**Protestant**	25	22	0.95(0.52, 1.73)	
Teaching category				
**1-4**^**th**^	116	119	1.00	1.00
**5-8**^**th**^	207	123	1.73(1.23, 2.42)	1.54(1.05, 2.24)**
Marital status*				
**Single**	76	43	1.00	
**Married**	204	154	0.75(0.49, 1.15)	
**Divorced**	25	31	1.46(0.24, 0.87)	
**Widowed**	18	14	0.73(0.33, 1.61)	
Family spec use*				
**No**	252	173	1.00	
**Yes**	71	69	0.71(0.48, 1.04)	
Age in years*				
**21–30 years**	99	56	1.00	1.00
**31–40 years**	59	47	0.71(0.43, 1.18)	1.56(0.57, 2.37)
**41–50 years**	56	51	0.62(0.38, 1.03)	1.61(0.64, 4.02)
**51–70 years**	109	88	0.70(0.46, 1.08)	3.05(1.07, 8.72)**
Years of experience				
**1–10 years**	94	44	1.00	1.00
**11–20 years**	85	66	0.60(0.37, 0.98)	0.63(0.30, 1.33)
**21–30 years**	75	60	0.59(0.36, 0.95)	0.41(0.15, 1.14)
**31–40 years**	69	72	0.45(0.28, 0.73)	0.23(0.07, 0.72)**

* Non-significant,

** P-value <0.05,

*** p<0.001

COR = Crude odds ratio, AOR = Adjusted odds ratio

As we can note from Table 4 the odds of favorable attitude towards refractive error among study participants, being male was two times greater than the odds of favorable attitude among females [AOR = 2.03 (95% CI: 1.37, 3.01)]. The odds of favorable attitude towards refractive error among study participants aged 51–70 years were three times greater than the odds of favorable attitude for those subjects aged 21–30 years [AOR = 3.05 (95% CI: 1.07, 8.72)]. The odds of favorable attitude towards refractive error among subjects who had 31–40 years of experience were 4.35 times less than the odds of favorable attitude for those subjects who had 1–10 years of experience [AOR = 0.23 (95% CI: 0.07, 0.72)]. The odds of favorable attitude towards refractive error among subjects working in private schools were 1.76 times larger than the odds of favorable attitude among subjects working in government schools [AOR = 1.76 (95% CI: 1.06, 2.93)]. And also the odds of favorable attitude towards refractive error among subjects teaching under 5^th^-8^th^ category were 1.54 times greater than the odds of favorable attitude among subjects teaching under 1^st^-4^th^ category [AOR = 1.54 (95% CI: 1.05, 2.24)].

## Discussion

In this study, about 55.9% (95% CI: 51.9, 59.8) of primary school teachers had good knowledge regarding a refractive error in school children. This result is lower as compared to other studies in India (68%, 72%), Singapore (73.4%), Nigeria (66.9%), Ghana (82%), Saudi Arabia (88.99%) and Cambodia (66.7%) [16,18,20,21,25,26]. This discrepancy may be due to the difference in accessibility of eye care services and utilization. The other explanation may be a difference in the advancement of eye care services and the existence of eye health programs in schools in these countries, which can affect their knowledge about the refractive error [27].

The odds of good knowledge regarding refractive error among study participants who had the previous history of spectacle were two times greater than the odds of good knowledge for those subjects who didn’t use spectacle. This finding is also consistent with other research conducted in Singapore[18]. This association is due to the fact that one advantage of spectacle use is to correct refractive error and the whys of spectacle wear may be explained to patients before dispensing, so respondents may understand what refractive error is.

In addition to this the odds of good knowledge regarding refractive error among study participants with previous history of eye examination were 1.67 times higher than the odds of good knowledge for those subjects with no history of eye examination. This finding is in line with a study conducted in Singapore and India [17,18]. This might be due to the chance to get information and eye health education regarding refractive error while they are visiting eye care center for their ocular problems.

The odds of good knowledge regarding refractive error among study participants who had previous training on eye health were two times greater than the odds of good knowledge for study subjects with no history of training on eye health. This finding is agreed with other studies done in India and Nigeria [14,20]. This may be due to that training on eye health is inclusive of all important procedures and methods for identification of a refractive error in school children. In addition, most of the time training is delivered with a thorough practical session on how to test vision, how pupils with refractive error adjust themselves in the classroom and its possible consequence on education, in general, which raises knowledge level.

The odds of good knowledge regarding refractive error among subjects with 11–20 years of experience were 2.53 times higher than the odds of good knowledge among subjects who had 1–10 years of experience. This finding is agreed with a study done in India [16]. This is because of as experience increases exposure to eye conditions in student revealed for teachers and they can detect children’s eye conditions easily through experience.

In this study, about 57.2% (95% CI: 52.9, 61.4) of study participants had a favorable attitude towards a refractive error in school children. This finding is agreed with the study done in Cambodia [26]. But this finding is lower when compared with researches done in America and Ghana [21,28]. This may be due to the higher knowledge of elementary school teachers and easily accessible information and eye care providing centers in America and Ghana that can affect their attitude towards refractive error which can bring affirmative attitude[21,28].

The odds of favorable attitude towards refractive error among study participants aged 51–70 years were three times greater than the odds of favorable attitude for those subjects aged 21–30 years. This association is in line with a study done in Cambodia. A possible explanation could be that increasing age creates increased awareness about the refractive error, as older people are more likely to have a refractive error, which could bring an attitudinal shift to a favorable one.

The odds of favorable attitude towards refractive error among subjects who had 31–40 years of experience were 4.35 times less than the odds of favorable attitude for those subjects who had 1–10 years of experience. This may be because of those with 31–40 years of experience may have a refractive error that should be corrected, but unable to access spectacles to treat it, which may affect attitude towards refractive error.

The odds of favorable attitude towards refractive error among study participants, being male was two times greater than the odds of favorable attitude among females. The possible reason for this discrepancy could be socio-cultural effect on the community in which males may expose to information compared to females.

The odds of favorable attitude towards refractive error among subjects working in private schools were 1.76 times larger than the odds of favorable attitude among subjects working in government schools. This is due to good facilities such as access to the internet in private schools that can affect their attitude as compared to governmental schools. The other possible explanation could be study participants in private schools had a higher educational level (33% degree holder in private, 11.54% degree holder in government), which can affect their attitude.

The odds of favorable attitude towards refractive error among primary school teachers teaching under 5th-8th category were 1.54 times greater than the odds of favorable attitude among subjects teaching under 1^st^ -4th category. This may be due to education status of those teachers who are teaching under the category of 5^th^-8^th^ are higher than those teachers who are teaching under 1^st^– 4^th^ category, in which those with higher levels may be exposed to eye conditions and brought an attitudinal shift. In addition to those teachers under the category of 5^th^-8^th^ may realize the problems of their students’ vision, since it requires extensive effort due to its difficulty of performing well in this category or students may inform their vision problem to teachers. These all can bring a positive attitude towards refractive error.

## Conclusion

- Knowledge and attitude of study subjects towards refractive error were low.
- Therefore eye health education and training to primary school teachers directed towards bringing a significant change in the knowledge and attitude regarding refractive error must be stepped-up within eye health program.

### Ethical approval and consent to participate

The ethical approval was obtained from the school of medicine ethical review committee, University of Gondar. The aim of the study and its public health importance was described for each participant. The consent declared that participants’ participation is voluntary. It also described that there is no any risky through participating in this study. It was also clarified that they had full right to refuse from participating in this research and to withdraw at any time they wish. They would also have a full right to contact and ask the authors whenever they want. Finally, they were kindly requested to give their genuine response. If someone was willing to participate, we took his/her signature before the interview.

## Supporting information

S1 File Questionnaire and data extraction form to study knowledge, attitude and associated factors among primary school teachers towards refractive error in schoolchildren at Gondar city, Northwest Ethiopia, 2017. (PDF)

S2 File pre-test questionnaire. (PDF)

S3 File Cronbach's alpha result for attitude. (PDF)

S4 File Cronbach's alpha result for knowledge. (PDF)
