# Supplementary material for: Knowledge, attitude and associated factors among primary school teachers regarding refractive error in school children in Gondar city, Northwest Ethiopia
Source: PLoS One. 2018 Feb 15;13(2):e0191199. doi: 10.1371/journal.pone.0191199 (PMC5813908; doi:10.1371/journal.pone.0191199)
Supplement: S1 File — (PDF) [file pone.0191199.s001.pdf]

English version of data extraction format

Pre tested structured questionnaire for determining knowledge and attitude towards RE and associated factors among primary school teachers in Gondar city, Northwest Ethiopia.

Introduction

Good morning/afternoon, my name is -------------------------------- I am working for University of Gondar. I am a member of a research group working in GUH. I am studying knowledge, attitude and associated factors among primary school teachers towards refractive error in school children in Gondar city by asking questions. Your aqppropriate answers for all of our questions are important to know knowledge and attitude towards refractive error and associated factors. Your answers will be confidential and keep in secret. If you decide that, you do not want to participate in the study now or at any time in the future; it is your right not to participate in the study. But we appreciate if you participate and will take 20 minutes for us to complete the questionnaire. Thank you. Next, I will read a consent, which assures your interest to participate.

Do I have your permission to continue?

If yes thank you and continue --------------

If no, thank you and go to next study subject --------------

Data collector

Name ----------------------- signature ------------------------ date -------------------

Checked by supervisor

Name --------------------- signature--------------------- date-------------------

1. questions related to socio-demographic characteristics			
**S. N**	**Questions**	**Responses Category/answers**	**Remark**
1	Age in years	……………………	
2	Sex	1. Male 2. Female	
3	Religion	1. Orthodox 2. Muslim<br>3. Catholic 4. Protestant<br>5. Others specify	
4	Marital status	1. Single 2. Married<br>3. Divorced 4. widowed	
5	Monthly Income in birr	………………………	
**2. Education related factors**			
6	Educational status	1. Certificate 2. Diploma<br>3. Degree 4. Masters and above	
7	Year of experience in years	……………………..	
8	Major teaching category	1. 1st-4th 2. 5th -8th	
9	School type	1. Government 2. Private	
**3. Eye condition related factors**			
10	Spectacle use	1. Yes 2. No	
11	Duration of spectacle use in years	……………..	
12	Do you have eye examination before?	1. Yes 2. No	
**4. information related question**			
13	Did you have training on eye health before?	1. Yes 2. No	
14	Did any of your family use spectacle?	1. Yes 2. No	

P art II Knowledge related questions	
1. How do you describe refractive error?	A. An able to focus parallel rays of light coming from infinite<br>B. Reduction of vision<br>C. I don't know<br>D. Others specify……
2. What are symptoms of refractive error? (You can choose more than one answers).	
	A. Blurring of vision<br>B. Complaining of headaches<br>C. Rubbing eyes frequently<br>D. Avoiding close work<br>E. Others specify…………..
3. Refractive errors occur when	
	A. Eye infections are present<br>B. Nutritional deficiencies are present<br>C. Shape of eye prevent light rays from focusing<br>D. Others specify
4. Risk factor for refractive error include	
	A. Family history of refractive error<br>B. Contact with children with refractive error<br>C. Vitamin deficiency D.I don't know<br>E.Others specify……………..
5. Refractive error can	
	A. Not affect academic performance<br>B. Lead to poor academic performance<br>C. Improve the academic performance<br>D. I don't know<br>E. Other specify

6. Child with refractive error can develop	
	A. Squint<br>B. Lazy eye<br>C. I don’t know<br>D. Others specify…..
7. Refractive error affects	
	A. Only Near vision<br>B. Only Distant vision<br>C. Vision at different distances<br>D. I don’t know<br>E. Other specify…………….
8. Undetected refractive errors in childhood may lead to behavioral problems and adversely affect social interaction	
	A. Yes<br>B. No<br>C. I do not know
9. Can refractive error cause blindness	
	A. Yes<br>B. No<br>C. I don’t know
10. How can you detect refractive error	
	A. With retinoscope/eye examination<br>B. Visual acuity test<br>C. I do not know<br>D. Others specify
11. What are the correction means of refractive error other than spectacles?	
	A. Contact lens<br>B. Refractive surgery<br>C. Surgery<br>D. I don’t know<br>E. Others specify …………..

**Part III Questions related to Attitude towards refractive error**					
	Strongly agree	Agre e	neu tral	Disagr ee	Strongly disagree
12. Refraction in children should not be done only when the caregiver complains					
13. Refraction in children should not only done by an eye care provider.					
14. We can prevent the complications/impact of refractive error on children.					
15. Children can use spectacles effectively and comfortably.					
16. Refractive error cannot be treated with eye drops.					
17. Schoolchildren with visual impairment can attend school.					
18. Vision screening must be done when children start schooling.					
19. Improper use of correctly prescribed spectacles can impose damage on children.					
20. Did you use any of the following information to answer questions regarding refractive error?	1. Mass media<br>2.Heath personnel<br>3. Colleague<br>4. I didn’t use any information<br>5. Other specify…………………….				

Annex 5: Amharic version of data extraction format

የጎንደር ዩኒቨርሲቲ ህክምናና ጤናሳይንስ ኮሌጅ የዓይን ህክምና ክፍል

መለያ ቁጥር -----------------

የት/ቤቱ ስም------------------

<u>የመጠይቅ ቅፅ</u>

ጤናይስጥልኝ ------------- እባላለሁ፡፡የጎንደር ዩነቨርስቲ ሠተራተኛና የዩኑቨርስቲው የጥናት ቡድን አባል ነኝ፡፡በመነጸር የሚስተካከል አጥርቶ የማየት ችግርን በተመለከተ መምህራን ያላቸዉን እዉቀት፣ አመለካከትና ተያያዥ ምክንያቶችን በጎንደር ከተማ ውስጥ በሚገኙት አንደኛ ደረጃ መምህራን መካከል ቃለመጠይቅ በማድረግ እያጠናን እንገኛለን፡፡ይህ ጥናት እርስዎ በሚሰጡን መረጃ ላይ የተመሰረተ ስለሆነ ፍቃድዎ ከሆነ መረጃውን በመስጠት ትብብር እንዲያደርጉልን በትህትና እንጠይቃለን፡፡በጥናቱ ላይ መሳተፍ የማይፈልጉ ከሆነ አሁንም ሆነ በሂደት ወስጥ አለመስማማት ይችላሉ፡፡ሆኖም ግን ጥናቱ ከትንሽ ጊዜ መፍጀት ውጪ ምንም አይነት ጉዳት የማያመጣ ስለሆነ እንዲሳተፉ እናበረታተለን፡፡መረጃዎ ምስጢራዊነቱ የተጠበቀ ፣ለጥናቱ ብቻ የሚውልና ለሌላ ጉዳይ የማንጠቀምበት መሆኑን ልናረጋግጥልዎ እንወዳለን፡፡ቃለመጠይቁ 20 ደቂቃ የሚፈጅ ስለሆነ ፍቃደኝነትዎን በፊርማ እንዲያረጋግጡልንና ዉል እንዲወስዱልን በትህትና እየጠየቅን ወደ ቃለመጠይቁ እንሄዳለን፡፡

ለመሳተ ፍፈቃደኛ ከሆኑ ወደሚቀጥለው ገፅ ይለፉ.

ማንኛውም ሊያነሱ የሚፈልጉት ጥያቄ ካለዎት ተመራማሪውን በሚቀጥለው አድራሻ ማነጋገር ይችላሉ፡፡

ስም፡ዐቢይ ማሩ

ስ. ቁ፡ 0918182413

<u>መረጃውን የሰበሰበው</u>

ስም ---------------------------------- ፊርማ -------------------------- ቀን -------------------

<u>መረጃውን ያረጋገጠው</u>

ስም---------------------------------- ፊርማ -------------------------- ቀን ------------------

ሀ. ማኅበራዊ መረጃ በተመለከተ		
ተ.ቁ	ጥያቄ	የመልስ አማራጭ
1	ዕድሜ በዓመት	………………………
2	ፆታ	1. ወንድ 2. ሴት
3	የጋብቻ ሁኔታ	1. ያላገባ/ች 2. ያገባ/ች<br>3. የፈታ/ች 4. የሞተበት/ባት
4	ሀይማኖት	1. ኦርቶዶክስ 2. ሙስሊም<br>3. ፕሮቴስታንት 4. ካቶሊክ<br>5. ሌላ ካለ ይጥቀሱ……………………
5	ወርሀዊ ገቢ በብር	………………………
ለ. ትምህርታዊ ጉዳዮችን በተመለከተ		
6	የትምህርት ደረጃ	ሰርተፊኬት 2. ዲፕሎማ<br>3. ዲግሪ 4. ማስተርስና ከዚያ በላይ
7	ማስተማር ከጀመርሩ ስንት አመት ሆነዎት፣ በአመት ?	………………………
8	እያስተማሩበት ያለዉ የክፍል ደረጃ	1. ከ1-4ኛ ክፍል 2. ከ5-8ኛ ክፍል<br>3. ሌላ ካለ ይጥቀሱ………………………………
9	የሚያስተምሩበት ትምህርት ቤት አይነት	1.የግል 2.የመንግስት
ሐ. የአይን በሽታ በተመለከተ		
10	የአይን ምርመራ አድርገዉ ያዉቃሉ ወይ?	1. አወ 2. አላዉቅም
11	መነፀር ይጠቀማሉ?<br>መልስዎ አልጠቀምም ከሆነ ወደ ጥ.ቁ 13 ይሂዱ	1. እጠቀማለሁ 2. አልጠቀምም
12	መነፀር መጠቀም ከጀመሩ ስንት አመት ሆነዎት?	------------------
መ. መረጃን በተመለከተ		
13	የአይንን ጤና በተመለከተ ስልጠና ወስደዉ ያዉቃሉ	1. አዉቃለሁ 2. አላዉቅም
14	ከቤተሰብዎ መካከል መነጸር የሚጠቀም ሰዉ አለ?	1. አለ 2. የለም

መመሪያ፡- ይህ መጠይቅ በመነፀር የሚስተካከል አጥርቶ ስለማየት ችግር (sight problem) ያለዎትን እዉቀትና አመለካካት ለመለካት የተዘጋጀ ነዉ፡፡ እባክዎት እያንዳንዱን ጥያቄ በጥንቃቄ ካነበቡ በኋላ ከተዘረዘሩት ምርጫዎች ዉስጥ መልስ ይሆናል ያሉትን ያክብቡ፡፡

ክፍል 2 እዉቀትን በተመለከተ	
1. በመነፀር የሚስተካከል አጥርቶ የማየት ችግርን እንዴት ይገልጹታል?	1. የአይን ምስል ተሸካሚ ብርሀን ቦታዉ ላይ በትክክል አለማረፍ ነዉ<br>2. የአይን የማየት አቅም መቀነስ ነዉ 3. አላዉቅም<br>4. ሌላ ካለ ይጥቀሱ..
2. በመነፀር የሚስተካከል አጥርቶ የማየት ችግር ምልክቱቶች ምንድን ናቸዉ? (ከአንድ በላይ መምረጥ ይቻላል)	1. ብዥታ ያመጣል 2. ራስምታት ያመጣል<br>3. አይንን በተደጋጋሚ ማሸት 4. የቅርብ ስራዎችን ለመስራት መቸገር 5. አላዉቅም<br>6. ሌላ ካለ ይጥቀሱ..
3. በመነፀር የሚስተካከል አጥርቶ የማየት ችግር በምን ምክንያት ይከሰታል ብለዉ ያስባሉ?	1. በበሽታ አምጭ ህዋሳት ሲጠቃ (ኢንፌክሽን)<br>2. በምግብ እጥረት<br>3. የአይን ብርሀንን አብዝቶ (አሳንሶ) በመቀልበስ ግልጽ ምስል ባለመፈጠር<br>4. አላዉቅም<br>5. ሌላ ካለ ይጥቀሱ..................
4. በመነጸር ለሚስተካከል አጥርቶ የማየት ችግር ተጋላጭ የሚያደርገዉ ምን ሊሆን ይችላል?	1. ከቤተሰብ ተመሳሳይ ችግር ካለ<br>2. ችግሩ ካለባቸዉ ልጆች ጋር በመነካካት<br>3. የቫይታሚን ኤ እጥረት<br>4. አላዉቅም<br>5. ሌላ ካለ ይጥቀሱ........................
5. በመነጸር የሚስተካከል አጥርቶ የማየት ችግር በልጆች ት/ት ላይ ምን ሊያስከትል ይችላል?	1. በትምህርት ላይ ተፅዕኖ የለዉም<br>2. ለዝቅተኛ ዉጤት ይዳርጋል<br>3. በትምህርት ላይ መሻሻል እንዲኖር ይረዳል<br>4. አላዉቅም<br>5. ሌላ ካለ ይጥቀሱ.....................
6. በመነጸር የሚስተካከል አጥርቶ የማየት ችግር በልጆች እይታ ላይ ምን ሊያስከትል ይችላል?	1. የአይን መንሸዋረር 2. የአይን እይታ መስነፍ<br>3. አላዉቅም 4. ሌላ ካለ ይጥቀሱ....................
7. በመነጸር የሚስተካከል አጥርቶ የማየት ችግር የትኛዉን የንባብ ሁኔታ ሊያዉክ ይችላል?	1. ቅርብ ላይ ማንበብን 2. ርቀት ላይ የማንበብን<br>3. ሁሉም ርቀት ላይ በግልፅ የማየት ችግር 4. አላዉቅም<br>5. ሌላካለይጥቀሱ...................

8. በጊዜዉ ያልታከመ በመነጸር የሚስተካከል አጥርቶ የማየት ችግር በልጆች ላይ ባህሪያዊና ማህበራዊ ተፅዕኖ አለዉ::	1. እዉነት 2. ሀሰት 3. አላዉቅም
9. በመነጸር የሚስተካከል አጥርቶ የማየት ችግር አይነስዉረነትን ያስከትላል::	1. እዉነት 2. ሀሰት 3. አላዉቅም
10. በመነጸር የሚስተካከል አጥርቶ የማየት ችግር እንዳለ እንዴት ማወቅ ይቻላል?	1. በአይን ምርመራ መሳሪያ 2. የእይታ መጠንን በመለካት<br>3. አላዉቅም 4. ሌላካለይጥቀሱ................................
11. በመነፀር የሚስተካከል አጥርቶ የማየት ችግር ማከም የሚቻልበት ሌላ መንገድ ምን ሊሆን ይቻላል?	1. ብሌን ላይ በሚለጠፍ ሌንስ 2. በጨረራ ህክምና<br>3. በቀዶ ጥገና 4. አላዉቅም<br>5. ሌላ ካለ ይጥቀሱ...

ክፍል 3 አመለካከትን በተመለከተ

	በጣም እስማማለሁ	እስማማ ለሁ	ገለልተኛ	አልሰማ ማም	በጣም አልስማማም
12. ልጆች የአይን ምርመራ የሚደረግላቸዉ ወላጅ/ አሳዳጊ ሲጠይቅ ብቻ መሆን የለበትም::					
13. የልጆችን አይን ምርመራ ማድረግ ያለበት የአይን ጤና ባለሙያ ብቻ መሆን የለበትም፡፡					
14. በልጆች ላይ የሚከሰተዉን በመነፀር የሚስተካከል አጥርቶ የማየት ችግር የሚያደርሰዉን ጉዳት መከላከል ይቻላል፡፡					
15. ልጆች መነፀርን በአግባቡ መጠቀም ይችላሉ፡፡					
16. በመነፀር የሚስተካከል አጥርቶ የማየት ችግርን በአይን ጠብታ መድሀኒት ማከም አይቻልም::					
17. የእይታ ችግር ያለባቸዉ ተማሪዎች ትምህርት መከታተል ይችላሉ፡፡					
18. ልጆች ት/ት ሲጀምሩ የእይታ መጠን ልኬታ መደረግ አለበት፡፡					
19. ልጆች በሀኪም የታዘዘላቸዉን መነፀር በአግባቡ ካልተጠቀሙ እይታቸዉ ይጎዳል፡፡					

20. በመነፀር የሚስተካከል አጥርቶ የማየት ችግር በተመለከተ ያለዎትን መረጃ የት አገኙት? (ከአንድ በላይ መምረጥ ይቻላል)	ሀ. ከመገናኛ ብዙሀን<br>ለ. ከጤና ባለሙያ<br>ሐ. ከጓደኛ<br>መ. ሌላ ካለ ይጥቀሱ..........
