# Supplementary material for: Knowledge, attitude and associated factors among primary school teachers regarding refractive error in school children in Gondar city, Northwest Ethiopia
Source: PLoS One. 2018 Feb 15;13(2):e0191199. doi: 10.1371/journal.pone.0191199 (PMC5813908; doi:10.1371/journal.pone.0191199)
Supplement: S3 File — (PDF) [file pone.0191199.s003.pdf]

5.00	3.00	2.00	4.00	3.00	3.00	5.00	1.00
3.00	3.00	3.00	3.00	3.00	3.00	1.00	3.00
3.00	3.00	2.00	3.00	1.00	2.00	5.00	1.00
1.00	1.00	2.00	1.00	3.00	2.00	2.00	3.00
5.00	5.00	5.00	1.00	5.00	5.00	3.00	5.00
3.00	1.00	3.00	4.00	4.00	4.00	4.00	4.00
5.00	1.00	1.00	4.00	4.00	2.00	4.00	3.00
4.00	5.00	2.00	2.00	2.00	3.00	3.00	2.00
3.00	3.00	3.00	3.00	3.00	3.00	2.00	3.00
5.00	5.00	5.00	5.00	2.00	2.00	4.00	3.00
1.00	1.00	2.00	3.00	5.00	4.00	4.00	3.00
1.00	4.00	2.00	2.00	5.00	4.00	4.00	3.00
3.00	3.00	3.00	1.00	5.00	4.00	4.00	3.00
4.00	2.00	4.00	4.00	2.00	2.00	3.00	3.00
5.00	4.00	2.00	2.00	3.00	2.00	2.00	3.00
2.00	4.00	2.00	2.00	2.00	4.00	3.00	3.00
5.00	4.00	2.00	2.00	4.00	3.00	3.00	3.00
3.00	2.00	2.00	2.00	2.00	3.00	1.00	3.00
3.00	3.00	3.00	3.00	3.00	3.00	3.00	2.00
2.00	3.00	3.00	3.00	2.00	2.00	2.00	2.00
4.00	4.00	2.00	2.00	4.00	4.00	4.00	4.00
1.00	1.00	1.00	3.00	4.00	2.00	2.00	3.00
3.00	2.00	1.00	2.00	3.00	2.00	3.00	1.00
1.00	2.00	2.00	2.00	2.00	2.00	2.00	1.00
5.00	5.00	5.00	5.00	5.00	5.00	1.00	3.00
1.00	1.00	2.00	2.00	1.00	3.00	3.00	3.00
3.00	2.00	2.00	2.00	1.00	3.00	1.00	3.00
4.00	4.00	4.00	4.00	4.00	3.00	3.00	3.00
2.00	4.00	4.00	4.00	4.00	4.00	4.00	4.00
